# Potential Use of γδ T Cell-Based Vaccines in Cancer Immunotherapy

**DOI:** 10.3389/fimmu.2014.00512

**Published:** 2014-10-21

**Authors:** Mohd Wajid A. Khan, Matthias Eberl, Bernhard Moser

**Affiliations:** ^1^Institute of Infection and Immunity, School of Medicine, Cardiff University, Cardiff, UK

**Keywords:** immunotherapy, γδ T cells, cancer, antigen-presenting cells, vaccine

## Abstract

Immunotherapy is a fast advancing methodology involving one of two approaches: (1) compounds targeting immune checkpoints and (2) cellular immunomodulators. The latter approach is still largely experimental and features *in vitro* generated, live immune effector cells, or antigen-presenting cells. γδ T cells are known for their efficient *in vitro* tumor killing activities. Consequently, many laboratories worldwide are currently testing the tumor killing function of γδ T cells in clinical trials. Reported benefits are modest; however, these studies have demonstrated that large γδ T-cell infusions were well tolerated. Here, we discuss the potential of using human γδ T cells not as effector cells but as a novel cellular vaccine for treatment of cancer patients. Antigen-presenting γδ T cells do not require to home to tumor tissues but, instead, need to interact with endogenous, tumor-specific αβ T cells in secondary lymphoid tissues. Newly mobilized effector αβ T cells are then thought to overcome the immune blockade by creating proinflammatory conditions fit for effector T-cell homing to and killing of tumor cells. Immunotherapy may include tumor antigen-loaded γδ T cells alone or in combination with immune checkpoint inhibitors.

## Human Blood γδ T Cells

Human blood γδ T cells constitute a minor subset (1–5%) of total T cells that is characterized by their T-cell antigen receptors (TCR) encoded by rearranged V-gamma and V-delta genes. Among blood γδ T cells, those expressing Vγ9Vδ2–TCR greatly outnumber other γδ T-cell subsets whereas in peripheral tissues Vγ9Vδ2+ T cells are relatively scarce. Their numbers are substantially increased in blood of patients suffering from various infectious diseases, indicating an involvement of Vγ9Vδ2^+^ T cells in antimicrobial immunity. Indeed, early work revealed that Vγ9Vδ2^+^ T cells are selective for a microbial non-peptide metabolite, (E)-4-hydroxy-3-methyl-but-2-enyl pyrophosphate (HMBPP), found in numerous commensal and pathogenic microbes, including bacteria, *Plasmodium* and *Toxoplasma* species but not in man (1, 2). The same type of γδ T cells also responds (albeit at >10^4^-fold higher concentrations) to the structurally related compound isopentenyl pyrophosphate (IPP), a metabolite of the mevalonate pathway in eukaryotes including man. Of interest, IPP was suggested to be increased in stressed cells, such as tumor cells, and its interaction with a specific IPP- (and HMBPP-) binding protein (BTN3A/CD277) leads to Vγ9Vδ2^+^ T-cell activation (reviewed by the groups of E. Adams, E. Scotet, and T. Hermann in this Research Topic). IPP levels can be elevated artificially by addition of amino-bisphosphonates (such as zoledronate; see below) that inhibit an intracellular IPP-metabolizing enzyme. Consequently, treatment of PBMC with zoledronate results in the selective activation and outgrowth of Vγ9Vδ2^+^ T cells. The exquisite selectivity for HMBPP/IPP distinguishes Vγ9Vδ2^+^ T cells from αβ T cells that recognize a myriad of short peptides under MHC restriction. In fact, the Vγ9Vδ2–TCR endows a sizable army of blood γδ T cells to become immediately mobilized in response to a single class (“phosphoantigens”) of danger signals produced by microbes and possibly tumor cells (3). Relevant to the discussion below, this “mono-selectivity” allowed us to study the TCR-mediated functionality and migration properties of the entire Vγ9Vδ2^+^ T-cell population.

## Discovery of γδT-APC: γδ T Cells with Antigen-Presentation Function

The myriad of effector and memory T cells present in our body can be distinguished based on their functional and migratory profiles. In fact, the characteristic functions of individual T cells are intimately related to their migratory properties, as exemplified by the distinct chemokine receptor profiles decorating individual T helper subsets (4, 5). For example, the chemokine receptor CXCR5 identifies follicular B helper T (T_FH_) cells that are specialized in orchestrating T cell-dependent antibody responses within the follicular compartments of secondary lymphoid tissues (6, 7). Our research on human blood γδ T cells (Vγ9Vδ2-TCR^+^ γδ T cells) began with the realization that treatment of γδ T cells with phosphoantigens induced the rapid and transient expression of CCR7 (8), the chemokine receptor enabling the rendezvous between naïve/central memory T cells and mature DC within lymph nodes (9). Immunological analyses revealed their presence in the T-cell zone but also B-cell follicles, the latter location suggesting that γδ T cells affect humoral responses. Indeed, and similar to T_FH_ cells, co-culture of γδ T cells with tonsillar B cells resulted in massive production of antibodies (8). In support, another lab identified CXCR5 on activated γδ T cells (10) and, recently, we and others reported the expression of the IL-21 receptor, linking follicular γδ T cells with T_FH_ cells and their B-cell targets (11, 12).

CCR7 expression is also in line with the view that activated γδ T cells team up with αβ T cells and/or DC in the T-cell compartment of secondary lymphoid tissues. In fact, short-term (1–3 days) activation of γδ T cells with the phosphoantigens IPP or HMBPP resulted in *de novo* expression (or up-regulation) of cell surface proteins normally associated with DC, including antigen presentation (MHC class I and II), co-stimulatory (CD40, CD80, CD86), and adhesion (CD11a, CD11b, CD11c, CD18, CD54) receptors. This observation led to detailed investigations into the possibility that activated (CCR7^+^) γδ T cells behaved like *bona fide* antigen-presenting cells (APC). Indeed, short-term activated γδ T cells were capable of processing simple (tetanus toxoid) and complex (*M. tuberculosis* PPD) protein antigens and inducing antigen-specific immune responses in primary, autologous αβ T cells (13). Activated γδ T cells did this equally well as donor matched monocyte-derived DC (moDC). Of note, short-term activated αβ T cells failed to express DC markers and were unable to present peptide antigens to αβ T cells, although under special conditions cloned T-cell lines were reported to induce responses in antigen-specific responder T-cell lines (14). Subsequent studies revealed that short-term activated γδ T cells were also capable of antigen cross-presentation, a process describing the induction of CD8^+^ αβ T-cell responses to extracellular antigens. The precise mechanisms underlying antigen cross-presentation require further clarification, although it is generally assumed that extracellular antigens need to overcome intracellular membrane barriers in order to access the proteasome in the cytoplasm, which produces the peptide substrates for loading onto nascent MHC class I molecules (15). This process is in clear contrast to the classical MHC I pathway involving the processing of endogenous self- or microbe-derived antigens. Obviously, CD8^+^ T cells-mediated killing of tumor and infected cells depends on cross-presentation capabilities of DC involved in processing corresponding cell debris. Activated γδ T cells are excellent cross-presenting APC, as shown by us (16, 17) and another laboratory (18). In fact, they seem to be able to do so more consistently than donor matched moDC, which may be explained by the reduced lysosomal activity in γδ T cells (17). After all, being a subtype of phagocytes, DC are very efficient in antigen uptake and intracellular degradation. Of note, γδ T cells were shown to phagocytose opsonized particles (*E. coli*, cell debris, microbeads), suggesting that phagocytosis, in addition to macropinocytosis, can contribute to antigen processing in this novel type of APC (18, 19). Induction of CD8^+^ T-cell responses by peptide-pulsed γδ T cells was also demonstrated by another group (20, 21). Finally, activated γδ T cells resemble professional APC in their ability to trigger antigen-specific responses in naïve (antigen inexperienced) T cells. In summary, all these findings document unexpected properties that are normally associated DC and prompted us to designate activated (but not freshly isolated, resting) Vγ9Vδ2^+^ T cells as γδT-APC (1).

In the past, γδ T cells have been portrayed as uniquely situated at the cross-road between innate and adaptive immunity, bridging both worlds by means of expressing recombined TCR involved in γδ T-cell activation and target cell lysis (hallmarks of adaptive immunity) while at the same time recognizing non-peptide antigens (phosphoantigens) in an MHC-unrestricted fashion similar to pattern recognition receptors (hallmarks of innate cells) (22, 23). Our findings with γδT-APC add to this functional dichotomy, although proof of APC functions under *in vivo* conditions is still missing. Because γδ T cells respond to stress, e.g., elevated levels of IPP in tumor cells and microbes harboring HMBPP, one could envisage that the phosphoantigen-induced killing of target cells by mobilized γδ T cells is accompanied by the processing of tumor/microbial antigens and the subsequent induction of αβ T-cell responses. Activated γδ T cells are known for their abundant secretion of proinflammatory cytokines (TNFα, IFNγ) and, accordingly, γδT-APC-mediated differentiation of CD4^+^ αβ T cells resulted in effector cells dominated by a Th1-type cytokine profile (13). It will be important to see if under appropriate co-stimulatory conditions, γδT-APC are also capable of generating alternative Th subsets. It is worth mentioning, however, that γδT-APC are not equipped with the plethora of receptors sensing environmental cues as DC and, thus, are unlikely to compete with the numerous highly specialized DC whose inherent function it is to control T-cell functions in all aspects of immunity (health and disease). Instead, it appears that the APC function of γδ T cells is limited to situations where they become activated by their TCR ligands (phosphoantigens).

## Methods for the Generation of Antigen-Presenting γδT-APC

Autologous moDC have been widely used in clinical trials as cellular vaccine with limited success. In fact, despite >10 years of translational research, clinically approved, moDC-based therapies still do not exist. Bottlenecks are manifold, including the difficulty of obtaining large numbers of moDC with robust APC function. Our recent findings suggest that γδT-APC represent a promising alternative to moDC. Many laboratories have already demonstrated how easy it is to expand γδ T cells to very large numbers during *in vitro* culture (24–35). Based on these findings, we have developed a method for the generation of *in vitro* expanded γδT-APC that may provide the basis for their large-scale manufacture under clinical-grade conditions (36). In brief, best results, both in terms of yield and quality, are achieved when PBMC samples isolated from fresh blood are stimulated once with zoledronate and then cultured in the presence of the growth factors IL-2 and IL-15. Zoledronate is a bisphosphonate drug known for its ability to increase intracellular IPP in target cells to levels sufficient for the selective activation of Vγ9Vδ2^+^ γδ T cells (37). After 14 days of culture, γδTells have expanded >1000-fold and >800-fold with PBMC of healthy individuals and melanoma patients, respectively, yielding 10–50 million γδ T cells per milliliter of whole blood. Day 14 γδ T cells retained their functionality as assessed by cytokine secretion and proliferation in response to HMBPP. Secreted cytokines included IFNγ and TNFα, which are typically produced by primary γδ T cells in response to phosphoantigens, as well as several inflammatory chemokines whereas immune inhibitory cytokines (TGFβ, IL-10) were not detected. Day 14 γδ T cells also killed tumor cells during *in vitro* culture. Importantly, and similar to short-term activated γδ T cells, expanded γδ T cells largely retained many cell surface receptors normally associated with APC, including antigen presentation (MHC I and II), co-stimulation (CD80, CD86), and adhesion (CD11a, CD54) molecules. Some of these were further elevated or re-expressed transiently (CD40 and CCR7) in response to re-stimulation with HMBPP. APC marker expression was in line with their functionality. Without need for re-stimulation, day 14 γδ T cells were able to process simple (influenza M1) and complex (PPD) antigens and to induce antigen-specific αβ T-cell responses in both cultured responder cell lines and primary responder cells present in unfractionated PBMC. Stimulation with HMBPP did not further enhance their APC function although, as discussed earlier, expanded γδ T cells still largely maintained their responsiveness (proliferation, cytokine secretion) to phosphoantigens. In summary, the current state of our research documents the feasibility of transforming peripheral blood γδ T cells into almost unlimited numbers of antigen-presenting γδT-APC. We now need to investigate the ability of day 14 γδ T cells to process tumor antigens, either in purified form or as tumor cell extracts. We believe that these APC are able to do so since we have already shown that activated γδ T cells efficiently processed extracts of influenza-infected epithelial cells and induced M1-specific αβ T-cell responses (17). It is further possible that interaction of γδ T cells with tumor cells not only leads to tumor cell killing but also to tumor antigen-presenting γδT-APC able to induce αβ T-cell responses locally (tumor tissue) or at distal sites (lymph nodes).

## A Protocol for a First-in-Man Clinical Trial with Tumor Antigen-Presenting γδT-APC

**Figure 1:**
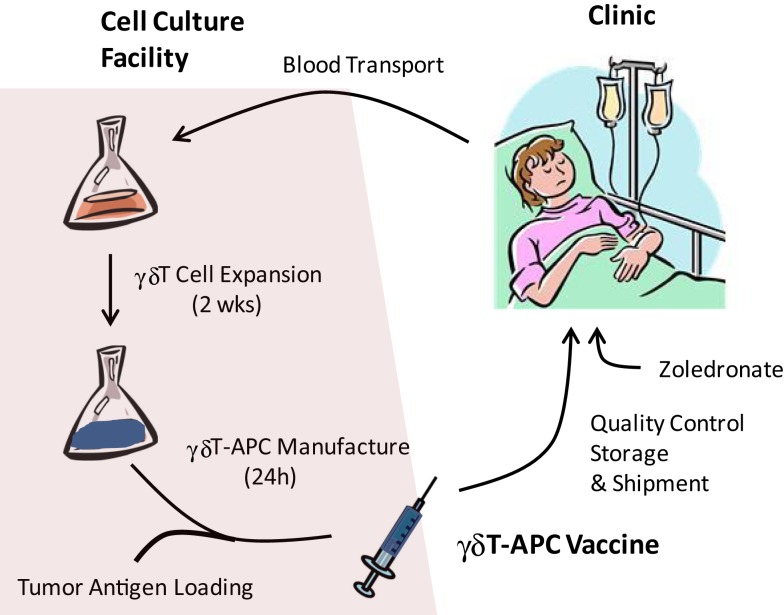
**Manufacture of tumor antigen-loaded γδ T-APC for immunotherapy**. Venous blood samples from cancer patients are shipped to the centralized γδ T-APC manufacture facility. Isolated PBMC are stimulated with single doses of zoledronate and expanded during *in vitro* culture for 2 weeks in the presence of IL-2 and IL-15. Expanded γδ T cells are treated for 24 h with tumor antigen (purified proteins, cell extracts) or, in cases of low purity, enriched by magnetic beads isolation prior to tumor antigen treatment. After reformulation, the γδ T-APC vaccine undergoes quality control tests and is frozen for transport to the clinic. γδ T-APC vaccine infusions may be followed by i.v. injections of zoledronate in order to trigger γδ T-cell responses (cytokine secretion, lymph node homing).

Current state of research supports the following protocol for a first-in-man clinical trial with tumor antigen-presenting γδT-APC (Figure 1). Preliminary data indicate anticoagulant-treated whole blood survives a 24 h shipment period in that PBMC samples retain responsiveness to zoledronate and IL-1/IL-15; similarly, we know that tumor antigen-loaded γδT-APC can be frozen for storage and/or shipment to cancer clinics. Therefore, it appears that cancer patients will not need to visit a centralized γδ T-cell culture facility for donating blood samples and receiving γδ T cell-based vaccine infusions. A simplified procedure of γδT-APC handling and distribution will facilitate clinical trials involving multiple centers. Following 2 weeks on *in vitro* culture as described above, tumor antigen-loaded γδT-APC will be prepared by culturing expanded γδ T cells for 24 h in the presence of defined tumor antigen(s) or tumor cell extracts. A personalized immunotherapy protocol will involve the treatment autologous γδT-cell preparations with extracts from the patient’s own tumor cells. After washing and reformulation, tumor antigen-loaded γδT-APC will be divided into individual bolus samples and then frozen in liquid nitrogen for shipment to corresponding cancer clinics. The frozen tumor antigen-loaded γδT-APC samples will then be prepared locally for i.v. infusion into cancer patients according to treatment regimens that need to be defined during clinical trials. Ideally, a single round of cell culture will provide enough tumor antigen-loaded γδT-APC for carrying out the entire round of treatment, which may follow a prime-boost protocol. Obviously, effective cell doses need to be established before being able to apply arithmetic for the generation of expanded γδ T cells. A small sample of tumor antigen-loaded γδT-APC will be retained for quality control that will include sterility, purity, and APC phenotype assessments. Frequently, PBMC from cancer patients yield reduced numbers of expanded γδ T cells or cell preparations containing large (>50%) numbers of non-γδ T cells (mostly αβ T cells and/or NK cells) (36), suggesting that a single magnetic beads purification step could improve the vaccine function of antigen-loaded γδT-APC. Last but not least, outcome measures for the γδT-APC-based vaccine treatment need to be defined but will include immunological parameters determined in blood samples taken at intervals after each infusion step as well as clinical parameters (e.g., see details in EMA document)^1^. The above treatment protocol may be extended to include a single i.v. infusion of zoledronate 24 h after administration of each γδT-APC vaccine infusion. We know that expanded γδ T cells retain full functionality (36). Equally important, γδ T cells in cancer patients who have received γδ T-cell infusions and even endogenous γδ T cells of cancer patients who did not receive γδ T-cell infusions were reported to respond to i.v. injections with zoledronate (32, 33, 38–43). Zoledronate is a safe medication that is frequently used to treat patients with bone disorders (44). Therefore, zoledronate may induce proinflammatory cytokine (TNFα, IFNγ) production and lymph node homing receptor (CCR7) expression and, thus, enhance the vaccine effect in infused γδT-APC.

It is important to emphasize that γδT-APC-based vaccines target the immune system in cancer patients and beneficial effects could include tumor control through immediate mobilization of endogenous, tumor-specific effector T cells, or establishing tumor immunosurveillance involving the generation of long-lived, tumor-specific memory T cells (or a combination of both processes). In this regard, the here described method involving tumor antigen-loaded γδT-APC differs fundamentally from current clinical trials exploiting the tumor killing properties of expanded γδ T cells (24–35).

## Concluding Remarks

Many questions remain, including the one related to the type of patients selected for a first-in-man clinical trial. As mentioned above, γδT-APC-based vaccines target the patients’ own immune system, indicating that patients suffering from tumors with well described immunogenicity profiles would benefit the most (45). Of course, tumor-specific immunity is a subject of intensive investigations and numerous tumor antigens have been associated with certain types of tumors. However, we are still far from understanding the complex adaptive immune processes involved in steady-state tumor control (immunosurveillance) and in tumor progression (disease). Perhaps, the most promising indication relates to conditions with prominent involvement of inhibitory immune cells (Treg cells, myeloid-derived suppressor cells), and current success with immune checkpoint inhibitors [anti-CTLA-4 Abs (Ipilimumab), anti-PD-1 Abs (Nivolumab)] may point us in the right direction (46). In fact, combination therapy with γδT-APC-based vaccines and immune checkpoint inhibitors may even result in synergistic outcomes, i.e., blockade of inhibitory immune cells by immune checkpoint inhibitors may facilitate the stimulatory effect of γδT-APC-based vaccines leading to enhanced tumor-specific effector T-cell responses and long-lived immunosurveillance T-cell formation. T-cell responses against melanoma are well described (47), suggesting that melanoma patients are promising candidates for a first-in-man clinical trial with γδT-APC-based vaccines although other cancers with less well studied immunogenicity should not be excluded. We envisage that the proposed γδT-APC-based immunotherapy will not be restricted to a single type of cancer since the majority of human cancers are considered to evoke T-cell responses (45).

At present, a single cellular vaccine product (sipuleucel-T) showing limited clinical benefits for prostate cancer patients has been approved by the FDA. Thus far, DC-based vaccines have not developed beyond the experimental stage. We believe that our proposed γδT-APC-based therapy combines several features, including ease of manipulation and functional robustness that will make it a serious contender in the race to the first successful vaccine for use in immunotherapy of patients suffering from a broad range of cancers.

## Conflict of Interest Statement

The authors declare that the research was conducted in the absence of any commercial or financial relationships that could be construed as a potential conflict of interest.

## Footnotes

^1^http://www.ema.europa.eu/docs/en_GB/document_library/Scientific_guideline/2009/09/WC500003875.pdf
